# Guttiferone K suppresses cell motility and metastasis of hepatocellular carcinoma by restoring aberrantly reduced profilin 1

**DOI:** 10.18632/oncotarget.10992

**Published:** 2016-08-01

**Authors:** Kaikai Shen, Zhichao Xi, Jianling Xie, Hua Wang, Chanlu Xie, C.Soon Lee, Paul Fahey, Qihan Dong, Hongxi Xu

**Affiliations:** ^1^ School of Pharmacy, Shanghai University of Traditional Chinese Medicine, Shanghai, China; ^2^ Institute of Arthritis Research, Shanghai Academy of Chinese Medical Sciences, Guanghua Integrative Medicine Hospital/Shanghai University of T.C.M, Shanghai, China; ^3^ Engineering Research Center of Shanghai Colleges for TCM New Drug Discovery, Shanghai, China; ^4^ Nutrition and Metabolism, South Australian Health and Medical Research Institute, Adelaide, Australia; ^5^ Stanley Ho Center for Emerging Infectious Diseases, The Chinese University of Hong Kong, Hong Kong, China; ^6^ School of Science and Health, The University of Western Sydney, Sydney, Australia; ^7^ Central Clinical School and Bosch Institute, The University of Sydney, Sydney, Australia; ^8^ Department of Endocrinology, Royal Prince Alfred Hospital, Sydney, Australia

**Keywords:** hepatocellular carcinoma, cancer metastasis, Guttiferone K, profilin1, actin

## Abstract

Hepatocellular carcinoma (HCC) is an aggressive malignancy and the 5-year survival rate of advanced HCC is < 10%. Guttiferone K (GUTK) isolated from the *Garcinia* genus inhibited HCC cells migration and invasion *in vitro* and metastasis *in vivo* without apparent toxicity. Proteomic analysis revealed that actin-binding protein profilin 1 (PFN1) was markedly increased in the presence of GUTK. Over-expression of PFN1 mimicked the effect of GUTK on HCC cell motility and metastasis. The effect of GUTK on cell motility was diminished when PFN1 was over-expressed or silenced. Over-expression of PFN1 or incubation with GUTK decreased F-actin levels and the expression of proteins involved in actin nucleation, branching and polymerization. Moreover, a reduction of PFN1 protein levels was common in advanced human HCC and associated with poor survival rate. In conclusion, GUTK effectively suppresses the motility and metastasis of HCC cells mainly by restoration of aberrantly reduced PFN1 protein expression.

## INTRODUCTION

The incidence of hepatocellular carcinoma (HCC) has risen over the past 20 years, predominantly due to the increased incidence of chronic viral hepatitis, alcoholic abuse, and non-alcoholic steatohepatitis [1]. In 2012, HCC accounted for 7% of newly diagnosed cancer and caused 746,000 deaths worldwide [2]. Given that diabetes and obesity are risk factors for HCC [3, 4], it is envisaged that the incidence of HCC will remain high even with successful control of viral hepatitis infection through vaccination. Although surgery and transplantation can be curative for HCC, effective treatment for regional and distant metastatic HCC is limited. Based on the US National Cancer Institute's Surveillance, Epidemiology, and End Results database (SEER, 2003–2009), the 5-year relative survival rate of regional and distant metastatic HCC is 7% and 2%, respectively (www.cancer.org/cancer/livercancer/detailedguide/liver-cancer-survival-rates). At present, sorafenib is the only systemic treatment for patients with advanced stage HCC [1]. As a multi-kinase inhibitor that blocks the phosphorylation of RAF, VEGF, PDGF, and c-KIT targets, sorafenib increases the median survival and the time to radiologic progression by 3 months [5]. While encouraged by this newly established treatment for advanced HCC, novel therapeutic strategies to further improve the outcome are clearly needed.

*Garcinia* species are tropical evergreen trees and shrubs widely distributed in Southeastern Asia and used in folk medicine to promote detoxification and treat inflammation or wounds [6]. Caged xanthones, polycyclic polyprenylated acylphloroglucinols (PPAPs) and benzophenones belong to a family of Guttiferae and are the main bioactive components of the *Garcinia* genus. In recent years *Garcinia* species have been shown to possess anti-cancer properties [6–9]. Following the revelation that caged xanthones (e.g., gambogic acid) exhibit toxicity to both tumors [10] and organs including the liver and kidney [11], our research focus has been on the anti-cancer properties of PPAPs [12–14].

The present study describes the effect of Guttiferone K (GUTK), a bioactive PPAP found at high concentration in the fruits of *Garcinia yunnanensis* [15], on HCC cell migration and invasion *in vitro* and metastasis *in vivo*, as well as the molecular mechanisms by which GUTK exerts this action.

## RESULTS

### GUTK suppresses HCC cell motility and metastasis

**Figure 1:**
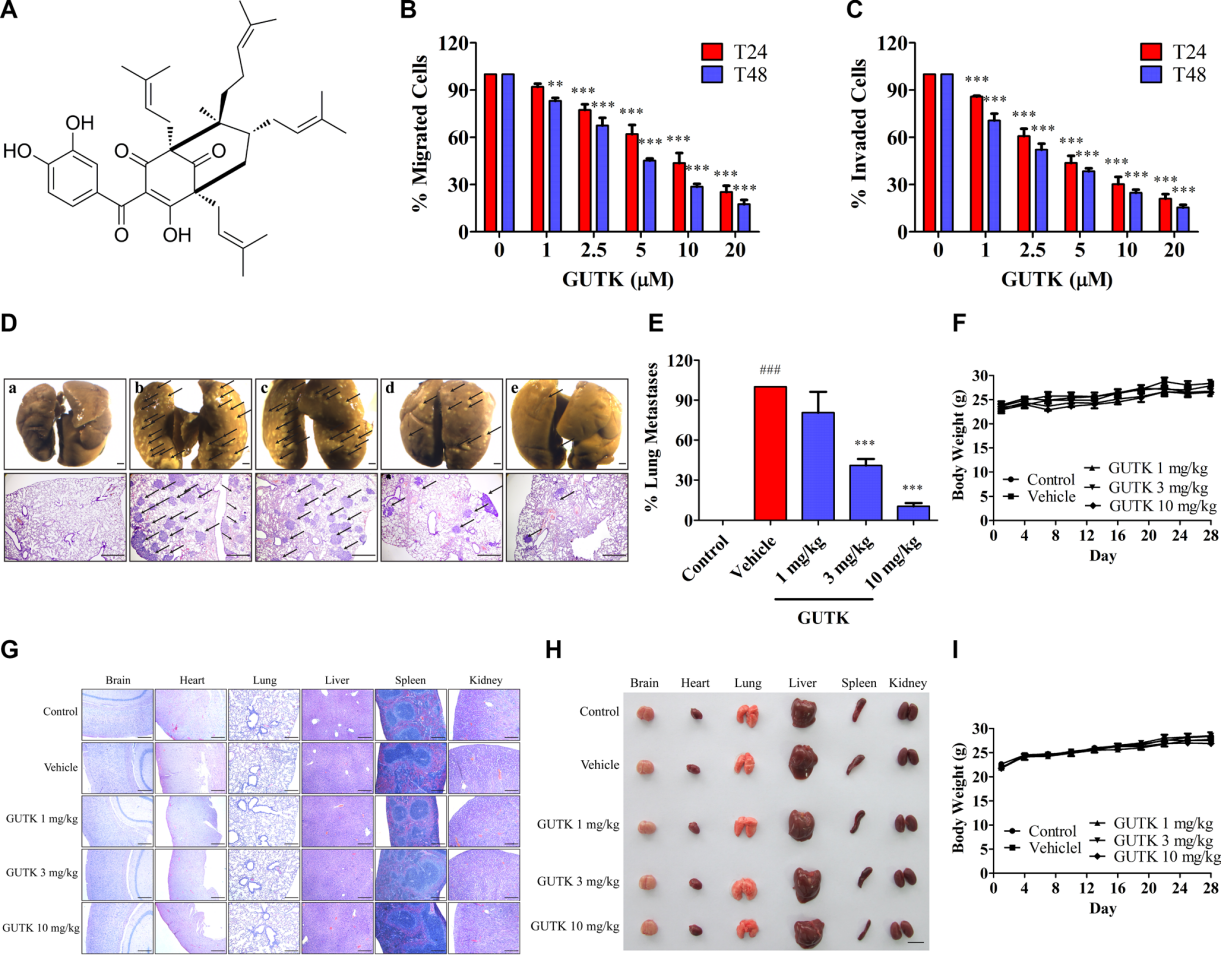
GUTK suppresses HCC cell motility and metastasis (**A**) Chemical structure of GUTK. (**B**, **C**) Cell migration and invasion were determined after incubation with GUTK (0–20 μM) in HepG2 cells for 24 h and 48 h. Data are shown as mean ± SEM; ^**^P < 0.01, ^***^P < 0.001 *vs*. control, *n* = 3. (**D**–**F**) After injected with HepG2 cells (1 × 10^6^ cells per mouse) *via* tail veins, BALB/c nude mice were *i.p*. injected with GUTK (1 mg/kg, 3 mg/kg, and 10 mg/kg) on the following and then every second day. On day 28, the mice were killed. Data were shown as mean ± SEM; *n* = 6 per group. (D) Upper panel: the lungs were fixed in Bouin's buffer and photographed. Lower panel: the lungs were fixed in 4% PFA, and sectioned for H&E staining. Arrow points to the tumor metastasis nodules. a: Control (No HepG2 cells injected); b: Vehicle (0.5% DMSO, 0.5% Tween 80 in PBS); c: GUTK 1 mg/kg; d: GUTK 3 mg/kg; e: GUTK 10 mg/kg. Scale bar = 1 mm. (E) Statistics of lung metastasis. ^###^*P* < 0.001, *vs*. Control; ^***^P < 0.001 *vs*. Vehicle. (F) The body weight recorded every three days. (**G**–**I**) BALB/c nude mice without HepG2 cell injection were randomly divided into 5 groups: Control (PBS), Vehicle (0.5% DMSO, 0.5% Tween 80 in PBS), GUTK treatment (1, 3, 10 mg/kg) every second day for 28 days *i.p*. (*n* = 8 per group). (G) H&E staining sections, scale bar = 1 mm. (H) Gross appearance in multiple organs, scale bar = 1 cm. (I) The body weight recorded every three days. Data are shown as mean ± SEM.

We isolated several compounds from the *Garcinia yunnanensis* and tested their effects on HCC cell motility. In a migration assay, one of these compounds known as GUTK (Figure 1A) reduced the motility of human hepatic cancer cells (HepG2, Li-7 and PLC/PRF/5) in a concentration- and time-dependent manner (Figure 1B and Supplementary Figure S1A and S1B). Likewise, GUTK suppressed cell invasion in the matrigel-coated transwell assay in HepG2, Li-7 and PLC/PRF/5 cells (Figure 1C and Supplementary Figure S1C and S1D). GUTK presented no cytotoxicity to HCC cells under the tested concentrations and duration (Supplementary Figure S2A, S2B and S1E–S1H).

To examine the effect of GUTK on HCC cell metastasis, we first performed liver orthotopic implantation with HepG2 cells in mice. However, there is no tumor nodule present in the tissues of brain, heart, lung, spleen and kidney (except of liver) as evidenced by hematoxylin-eosin staining in (Supplementary Figure S3). Therefore, we chose to use tail vein injection of HepG2 cells instead, and following administrated GUTK or the vehicle (0.5% DMSO, 0.5% Tween 80 in PBS) on every second day. After 28 days, the number of metastasized nodules in the lungs of mice treated with GUTK at 3 and 10 mg/kg was 59.1% and 89.4%, respectively, less than the vehicle group (Figure 1D and 1E). There was no difference in body weight between the vehicle and the GUTK-treated groups (Figure 1F). In mice with no HepG2 cell injection; there was no apparent change in cell morphology of vital organs and body weight among the untreated, the vehicle-treated, and the GUTK-treated groups (Figure 1G–1I). Taken together, GUTK is capable of inhibiting HCC cell migration, invasion and metastasis without apparent cytotoxicity.

### Profilin 1 (PFN1) mediates GUTK action on HCC cell motility

**Figure 2:**
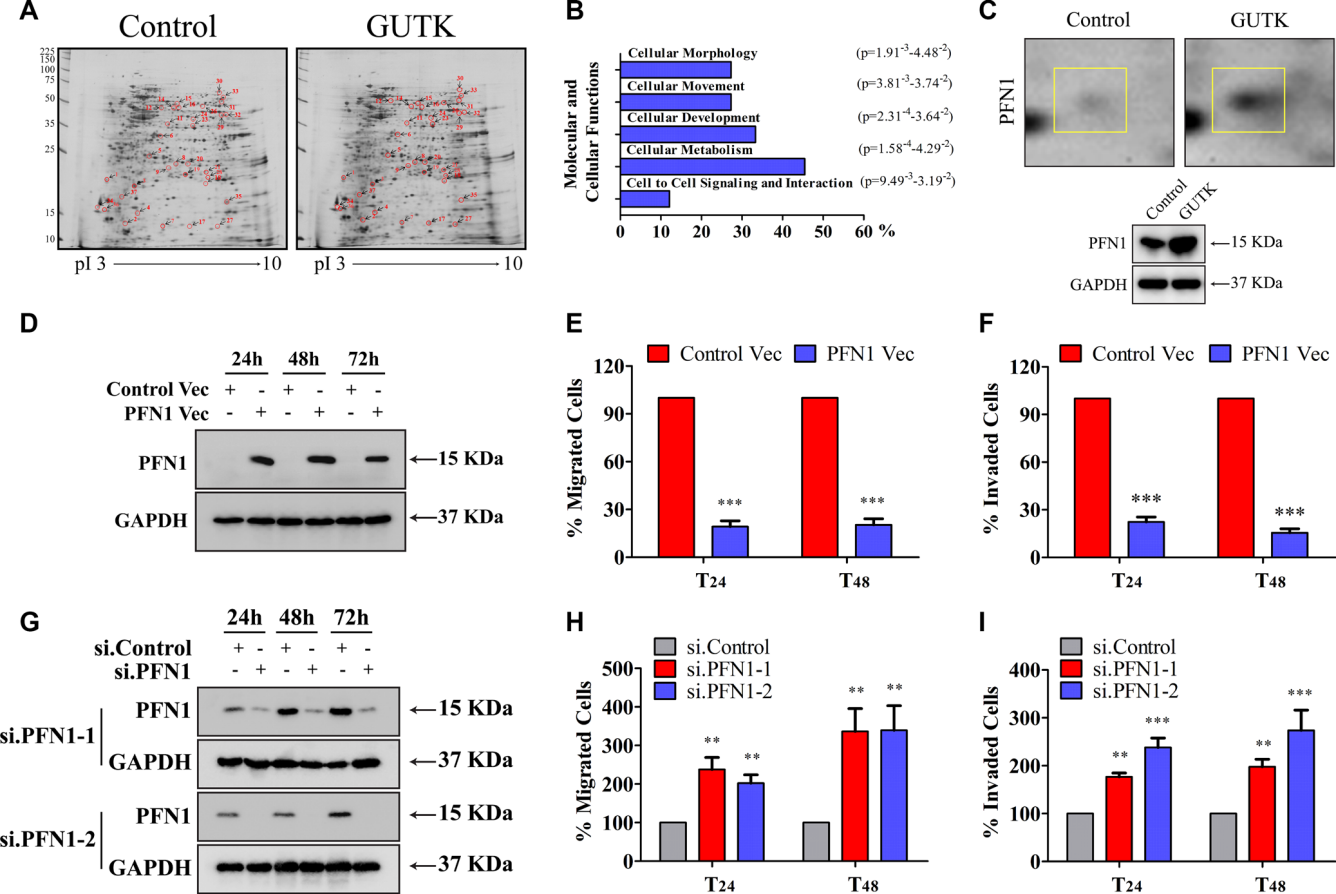
PFN 1 mediates GUTK action on HCC cell motility (**A**) Representative silver-stained-2-DE images of HepG2 cells treated with or without GUTK (20 μM) for 24 h. Differentially expressed spots are shown by the arrows. (**B**) Ingenuity pathway analysis (Ingenuity Systems, www.Ingenuity.com). Bars represent molecular and cellular functions that are significantly changed following incubation with GUTK. (**C**) Cropped and enlarged regions of the differently expressed PFN1 spot (top), and PFN1 protein expression in HepG2 cells treated with GUTK for 24 h by western blotting (bottom). (**D**) PFN1 in HepG2 cells transiently transfected with PFN1 or control vector for 24–72 h by western blotting. GAPDH served as a loading control. (**E**, **F**) The migration and invasion were determined in HepG2 cells transiently transfected with PFN1 or control vector for 24 h and 48 h. Data are shown as mean ± SEM; ^***^P < 0.001 *vs*. cells transfected with control vector. *n* = 3. (**G**) PFN1 in HepG2 cells transiently transfected with two individual PFN1 siRNAs (si.PFN1-1 and si.PFN1-2) or control siRNA (si.Control) for 24–72 h by western blotting. (**H**, **I**) The migration and invasion were determined in HepG2 cells transiently transfected with PFN1 siRNAs or control siRNA for 24 h and 48 h. Data are shown as mean ± SEM; ^**^P < 0.01, ^***^P < 0.001 *vs*. cells transfected with si.Control. *n* = 3.

To gain insight into the GUTK action, we compared the protein profiles of GUTK-treated with vehicle-treated HepG2 cells. Using two-dimensional gel followed by MALDI-TOF MS analyzes, we identified 33 proteins being altered (≥ three-fold) in GUTK-treated cells (Table 1); in which 21 were up- and 12 down-regulated. Ingenuity pathway analysis revealed that ~30% of the altered proteins fall into the functional class of “cellular movement” (Figure 2A and 2B). The protein PFN1 was up-regulated by 7.4 fold in the presence of GUTK (Figure 2C, upper panel). This was confirmed by western blotting (Figure 2C, lower panel). Since PFN1 functions as an actin-binding protein, we determined its potential in mediating GUTK action on cell motility.

**Table 1:** Differentially expressed proteins identified by 2-DE and MS analyses between the GUTKtreated and DMSO-treated HepG2 cells; 37 spots were selected for further MALDI-TOF-MS/MS-MS analyses, and 33 proteins were finally identified

Spot No.	Protein name	NCBI Accession No.	Theoretical molecular mass (KD)/PI	Peptide score (CI)	Fold change
1	Heterogeneous nuclear ribonucleoproteins C1/C2	P07910	33.7/4.95	222	6.0
2	ATP synthase subunit alpha	P25705	59.8/9.16	219	5.0
3	Dihydrolipoyllysine-residue succinyltransferase component of 2-oxoglutarate dehydrogenase complex, mitochondrial	P36957	49.1/9.11	604	5.9
4	Actin-related protein 2/3 complex subunit 5	O15511	16.3/5.47	321	−5.3
5	Cathepsin D	P07339	45.0/6.1	368	−3.0
6	60S acidic ribosoal protein P0	P05388	34.4/5.71	79	−5.0
7	Dermcidin	P81605	11.3/6.08	202	3.8
8	Metaxin-2	O75431	30.1/5.9	171	3.7
9	Phosphoglycerate kinase 1	P00558	45.0/8.3	690	4.2
11	Tubulin beta	P07437	50.1/4.78	501	−5.5
12	Glutathione synthetase	P48637	52.5/5.67	134	−6.0
14	Fibrinogen gamma chain	P02679	52.1/5.37	931	−4.0
15	Aldehyde dehydrogenase	P05091	56.9/6.63	100	5.0
16	Heterogeneous nuclear ribonucleoprotein H	P31943	49.5/5.89	232	4.6
17	40S ribosomal protein S12	P25398	14.9/6.81	655	52
18	Pyrroline-5-carboxylate reductase 1	P32322	33.6/7.18	199	4.5
19	Thioredoxin-dependent peroxide reductase, mitochondrial	P30048	28.0/7.67	329	4.4
20	Isopentenyl-diphosphate Delta-isomerase 1	Q13907	26.6/5.93	160	4.7
21	Heterogeneous nuclear ribonucleoprotein H	P31943	49.4/5.89	232	4.6
23	Protein disulfide-isomerase A6	Q15084	48.5/4.95	159	3.4
24	Elongation factor Tu	P49411	49.9/7.26	245	6.4
26	Aldehyde dehydrogenase X, mitochondrial	P30837	57.6/6.36	499	7.2
27	Profilin 1	P07737	15.2/8.44	326	7.4
28	Elongation factor Tu, mitochondrial	P49411	49.8/7.26	245	6.4
29	Acyl-coenzyme A thioesterase 1	Q86TX2	46.6/6.9	164	−4.3
30	Phosphoenolpyruvate carboxykinase [GTP], mitochondrial	Q16822	71.5/7.57	508	−5.9
31	UDP-glucose 6-dehydrogenase	O60701	55.7/6.73	332	−5.7
32	Fumarate hydratase	P07954	54.8/8.85	195	−3.7
33	ATP-dependent dihydroxyacetone kinase	Q3LXA3	59.3/7.12	225	−3.9
34	Dermcidin	P81605	11.3/6.08	202	3.8
35	Cofilin-2	Q9Y281	18.8/7.66	353	(−)
36	Protein disulfide-isomerase	P07237	57.4/4.76	810	(+)
37	Nascent polypeptide-associated complex subunit alpha	Q13765	23.4/4.52	1224	(+)

Firstly, we examined the effect of over-expression of PFN1 on HepG2 cell motility. Cells were also treated with sorafenib as a positive control (Supplementary Figure S4A–S4F), although sorafenib-treatment exhibits high cytotoxicity in HCC cells (Supplementary Figure S4G–S4L), which is more likely to be responsible for its anti-metastatic effects. Transient transfection of a PFN1 expression vector (Figure 2D) reduced HepG2 cell migration by 80% over 24–48 h compared to cells transfected with a control vector (Figure 2E). Over-expression of PFN1 similarly decreased the matrigel invasion (Figure 2F). The anti-migration and anti-invasion effects of PFN1 were further confirmed in pooled HepG2 cells in which over-expression of PFN1 was achieved by stable transfection of the expression vector (Supplementary Figure S5A–S5C). Likewise, overexpression of PFN1 decreased cell migration and invasion in Li-7 and PLC/PRF/5 cells (Supplementary Figure S6A–S6F).

Next, we examined the change in cell migration and invasion in HepG2 cells following the knock down of PFN1 by siRNA (Figure 2G). HepG2 cells had a 3 times higher rate of motility (Figure 2H) and invasiveness (Figure 2I) when PFN1 was markedly reduced. Silenced expression of PFN1 similarly induced cells migration and matrigel invasion in Li-7 and PLC/PRF/5 cells (Supplementary Figure S7A–S7F). We did not observe any change in cell death following transfection with the PFN1 vector or PFN1-specific siRNA in HepG2, Li-7 and PLC/PRF/5 cells (Supplementary Figures S2C, S2D, S6G, S6H, S7G and S7H).

**Figure 3:**
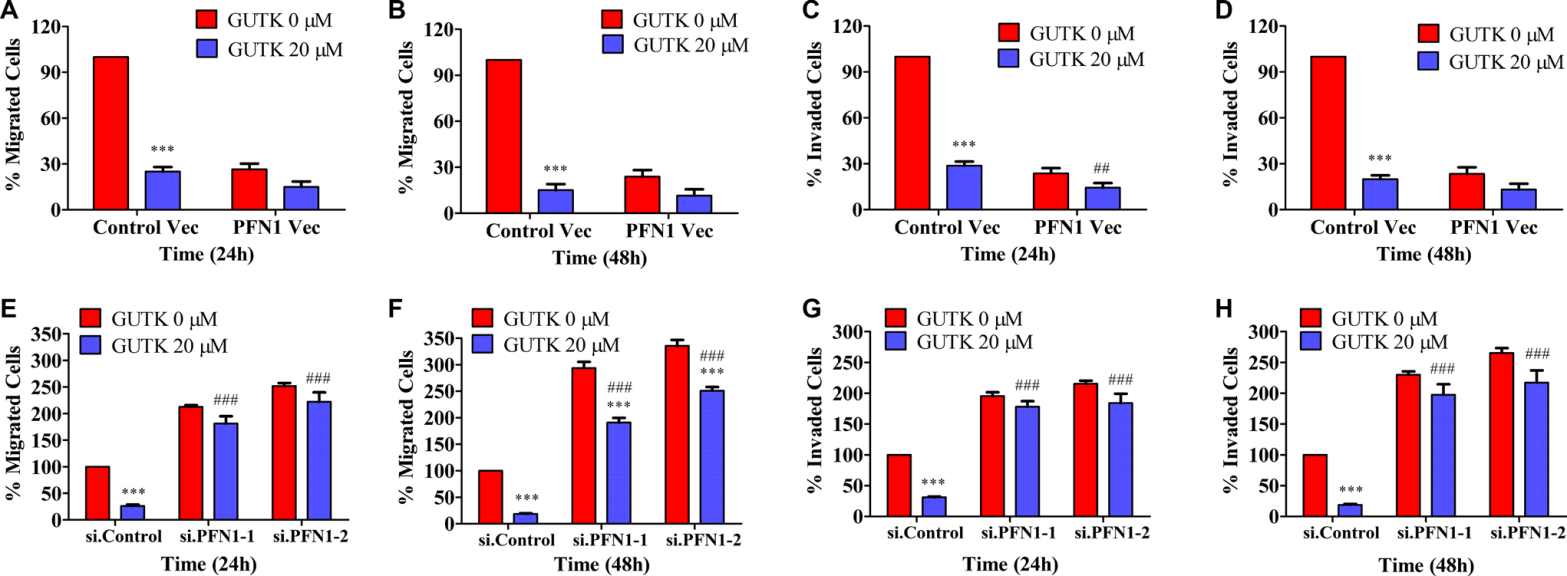
The effect of GUTK on HCC cell motility is diminished when PFN1 was either over-expressed or knocked-down (**A**, **B**) Cell migration was determined in GUTK-inhibited, PFN1-over-expressed HepG2 cells for 24 h and 48 h. (**C**, **D**) Cell invasion was determined in GUTK-inhibited, PFN1-overexpressed HepG2 cells for 24 h and 48 h. (**E**, **F**) Cell migration activity was determined in GUTK-inhibited, PFN1-knocked down HepG2 cells for 24 h and 48 h. (**G**, **H**) Cell invasion was determined in GUTK-inhibited, PFN1-knocked down HepG2 cells for 24 h and 48 h. Data are shown as mean ± SEM; ^***^P < 0.001 *vs*. GUTK 0 μM. ^##^*P* < 0.01, ^###^*P* < 0.001 *vs*. Control Vec or si. Control transfected cells treated with GUTK 20 μM. *n* = 3.

To verify that PFN1 could mediate GUTK action, we determined the effect of GUTK on the motility of HepG2 cells in which PFN1 was either over-expressed or knocked-down. By comparing with the parental HepG2 cells, GUTK had little inhibitory effect on the migration and invasion of HepG2 cells in which PFN1 was over-expressed (Figure 3A–3D) or knocked-down (Figure 3E–3H). These results demonstrate the biological significance of PFN1 and support the notion that GUTK suppresses HCC motility at least in part *via* the up-regulation of PFN1 expression.

### PFN1 mimics the effect of GUTK in suppressing HCC cell metastasis *in vivo*

**Figure 4:**
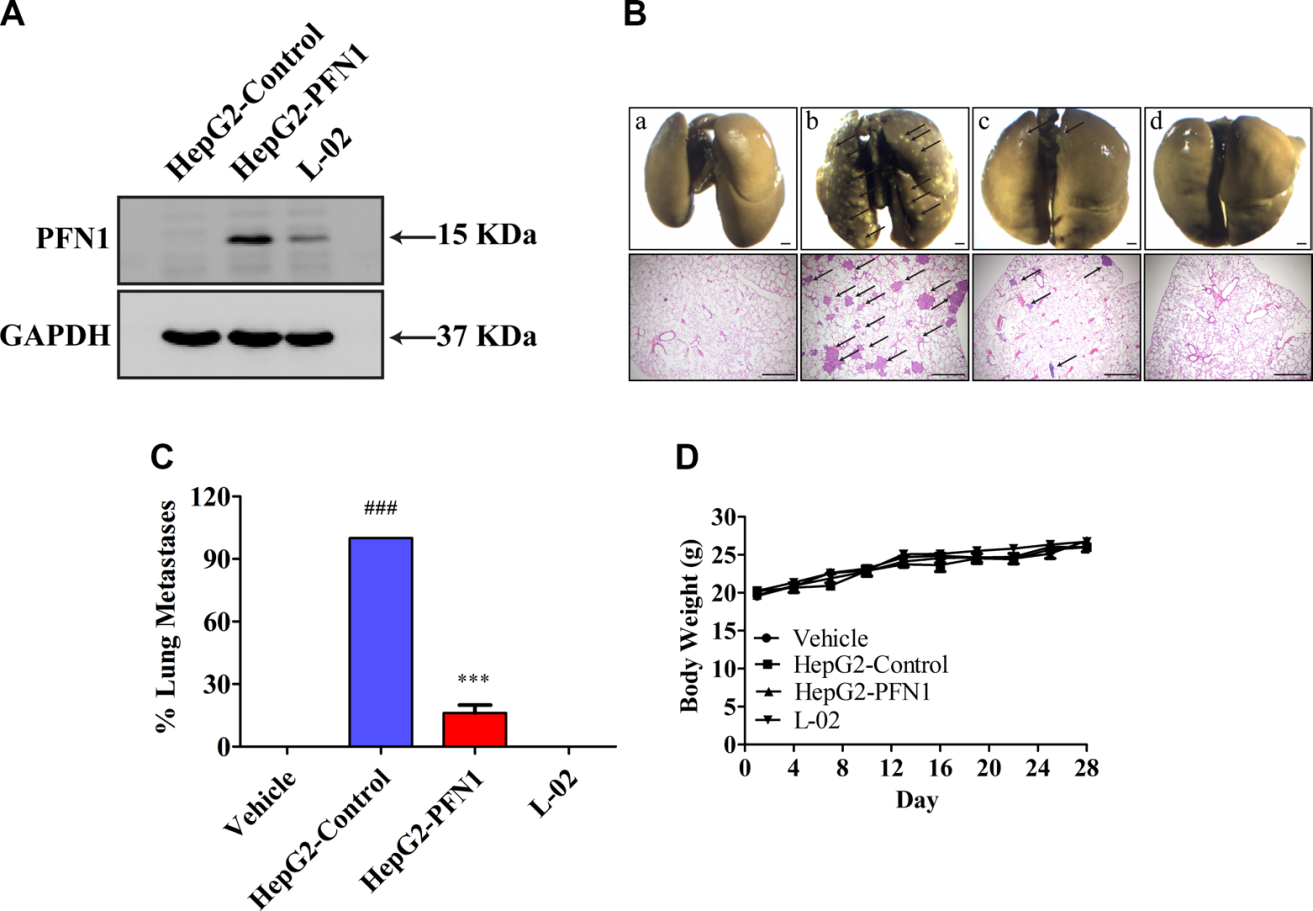
Restoration of PFN1 inhibits HCC cell metastasis (**A**) PFN1 protein expression in HepG2-Control, HepG2-PFN1, and L-02 cells by Western blotting. (**B**) After injection with HepG2-Control, HepG2-PFN1, and L-02 cells (1 × 10^6^ cells per mouse) *via* tail veins of BALB/c nude mice for 28 days. Upper panel: the lungs were fixed in Bouin's buffer and photographed. Lower panel: the lungs were fixed in 4% PFA, and sectioned for H&E staining. Arrow points to the metastasis nodules. a: Control (No HepG2 cells injected); b: HepG2-Control; c: HepG2-PFN1; d: L-02. Scale bar = 1 mm. (**C**) Statistics of lung metastasis. (**D**) The body weight was recorded every three days. Data are shown as mean ± SEM; ^###^*P* < 0.001 *vs*. Control; ^***^P < 0.001 *vs*. HepG2-Control. *n* = 8 per group.

To seek evidence that GUTK-induced PFN1 is necessary for the suppression of metastasis *in vivo*, we determined the effect of inducing PFN1 in HepG2 cells on lung metastasis in mice. At 28 days after injection *via* tail veins, mice injected with HepG2-Control cells (i.e., those stably transfected with a control vector, Figure 4A) developed extensive metastatic nodules in the lung as expected. However, mice injected with HepG2-PFN1 cells (i.e., those stably transfected with a PFN1 expressing vector, Figure 4A) exhibited either no or a few much smaller nodules (Figure 4B and 4C). As another control, mice injected with L-02 cells (a non-cancerous liver tissue derived cell line with high PFN1 expression) had no metastasized nodules in the lung (Figure 4B and 4C). There was no significant change in body weight in any group (Figure 4D). Hence, up-regulation of PFN1 expression alone in HepG2 cells is sufficient to suppress their metastasis *in vivo*.

### GUTK and PFN1 decrease actin filaments and proteins for actin polymerization in HCC cells

**Figure 5:**
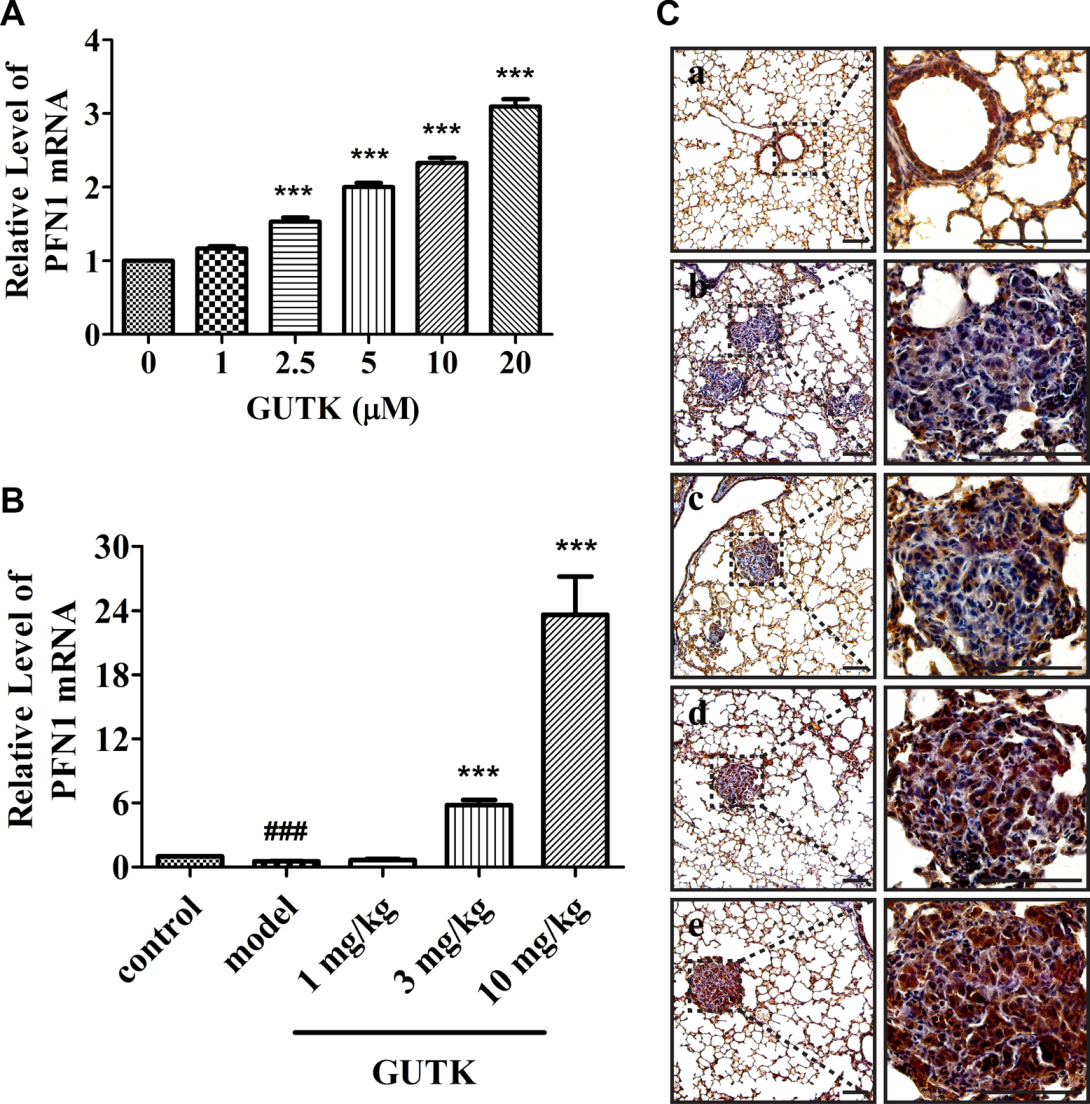
GUTK induces increases in the mRNA and protein expression levels of PFN1 *in vitro* and *in vivo* (**A**) mRNA expression levels of PFN1 and GAPDH were measured by qPCR after treatment with GUTK (0–20 μM) for 24 h in HepG2 cells. Data are shown as means ± SEM; ^***^P < 0.001 compared with control. *n* = 3 (**B**) mRNA expression levels of PFN1 and GAPDH were measured by qPCR in tumor sections of GUTK-treated mice. Data are shown as means ± SEM; ^###^*P* < 0.001, *vs*. Control; ^***^P < 0.001 *vs*. Vehicle. *n* = 6. (**C**) The expression of PFN1 was observed in tumor sections of GUTK-treated mice by immunohistochemistry assay. Scale bar = 100 μm. a: Control (No HepG2 cells injected); b: Vehicle (0.5% DMSO, 0.5% Tween 80 in PBS); c: GUTK 1 mg/kg; d: GUTK 3 mg/kg; e: GUTK 10 mg/kg. *n* = 6.

**Figure 6:**
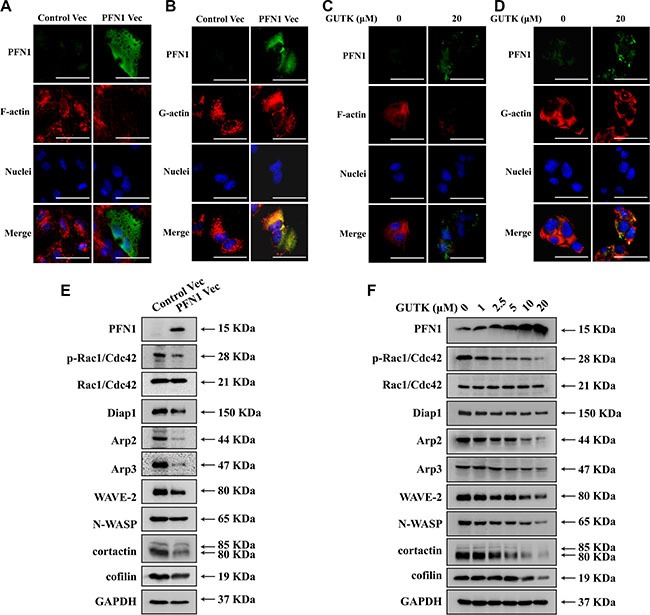
GUTK and PFN1 regulate key proteins involved in cell motility and metastasis (**A**) Staining of PFN1 (green), F-actin (phalloidin staining, red) and nucleus (blue) in HepG2 cells transiently transfected with control or PFN1 vector for 24 h. (**B**) Exactly same as (A) but with G-actin (red). Scale bar = 50 μm. (**C**, **D**) Same staining as (A) and (B) but treated with GUTK (0–20 μM). (**E**, **F**) Western blotting analysis for protein expression of PFN1, phospho-Rac1/Cdc42 at Ser^71^ (p-Rac1/Cdc42), Rac1/Cdc42, Diap1, Arp2, Arp3, WAVE-2, N-WASP, cofilin, cortactin and GAPDH in HepG2 cells transiently transfected with control or PFN1 vector for 24 h or treated with GUTK (0–20 μM) for 24 h. *n* = 3.

We observed an increase in the protein expression levels of PFN1 in response to GUTK in a concentration-dependent manner in HCC cells, including HepG2, Li-7 and PLC/PRF/5 cells (Figure 6F, Supplementary Figure S8A and S8B). We also monitored the mRNA levels of PFN1 in response to GUTK in these three HCC cell lines (HepG2, Li-7 and PLC/PRF/5) by qPCR. GUTK treatment led to an increase in the expression levels of PFN1 mRNA in all cell lines tested (increased by 3.1-, 1.9- and 2.2-fold, respectively) (Figure 5A and Supplementary Figure S8C and S8D). We harvested the lung tissue from control- and GUTK-treated mice to monitor the levels of PFN1 mRNA expression by qPCR as well as protein expression levels of PFN1 by immunohistochemistry. In agreement with the HCC cell data, there was an increase in the mRNA levels and protein expression levels of PFN1 in the lung tumor nodules from GUTK-treated mice in a dose-dependent manner (Figure 5B and 5C).

We explored the mechanisms by which PFN1 or GUTK suppresses HCC cell motility and metastasis. Over-expression of PFN1 (Figure 6A and 6B) or incubation with GUTK (Figure 6C and 6D) in HepG2 cells decreased the actin filaments (F-actin) determined by phalloidin staining. In contrast, the levels of monomer globular actin (G-actin), as detected by immunofluorescence staining, were not altered when PFN1 was over-expressed (Figure 6B) or incubated with GUTK (Figure 6D). We then examined the response of Rho-GTPase–dependent Arp2/3 complex and independent Diap1 proteins to PFN1 or GUTK. Arp2/3 protein expression was reduced in HepG2 cells when PFN1 was over-expressed (Figure 6E) or incubated with GUTK (Figure 6F). Concomitantly, phosphorylated Rac1/Cdc42, WAVE-2, N-WASP and cortactin were all decreased following over-expression of PFN1 (Figure 6E) or incubation with GUTK (Figure 6F). Diap1 was decreased in HepG2 cells when PFN1 was over-expressed (Figure 6E) but there was no obvious change in the presence of GUTK (Figure 6F). Cofilin was decreased in HepG2 cells after treatment with GUTK (Figure 6F) but there was no obvious change when PFN1 was over-expressed (Figure 6E). Therefore, the increase in PFN1 expression induced by GUTK treatment highly coincides with a reduction in the expression of tumor metastasis markers.

### Aberrantly reduced PFN1 expression in advanced HCC predicts poor survival

**Figure 7:**
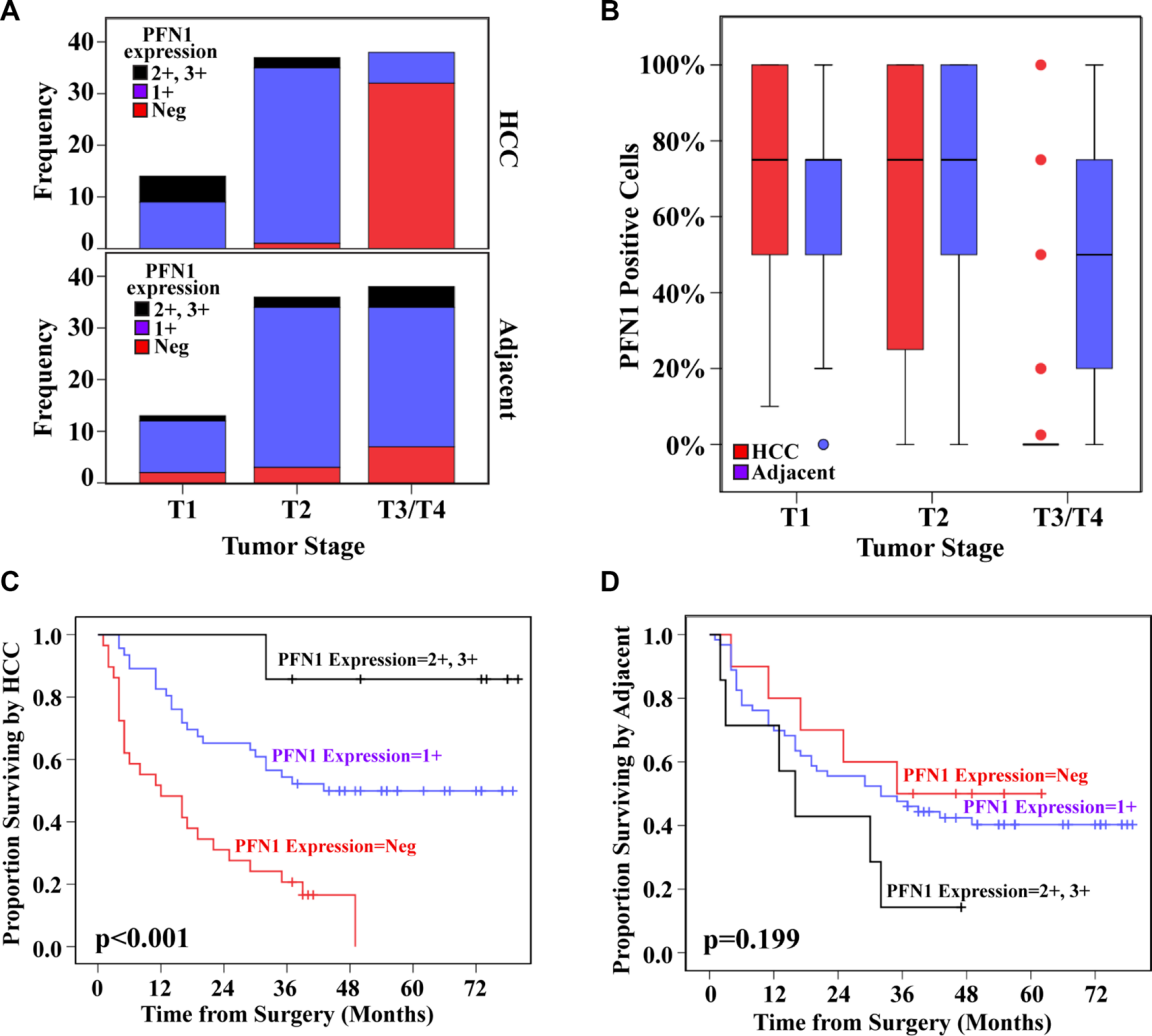
PFN1 expression levels and fraction of PFN1 positive cells in advanced HCC predict metastasis-free survival PFN1 expression level (**A**) and PFN1 protein positive cells (**B**) by tumor stage in HCC and adjacent tissue, respectively. Kaplan-Meier curves of survival by PFN1 expression in HCC tissue (**C**) and in adjacent tissue (**D**). The TMA contains 90 individual paired HCC and adjacent tissues, in which 86 were included in the analysis and the remaining four were unscorable due to tissue quality.

To evaluate the relevance of the reduced PFN1 to human HCC, we examined PFN1 expression in 86 individual HCCs and their adjacent non-cancer tissue specimens (Table 2) by immunohistochemical analysis. We noted significant reductions in the PFN1 protein levels (comparing negative staining against 1+, 2+ and 3+ combined, Figure 7A) or the percentage of PFN1 positive cells (Figure 7B) in later stage (T3 and T4) HCC, compared to their own adjacent non-cancer tissues. No similar difference was found in earlier stage (T1 and T2) HCC tissue. Moreover, low PFN1 levels or the fraction of PFN1 positive cells were associated with poor disease-free survival rate (Figure 7C and 7D). We also examined PFN1 expression levels in five liver cancer lines and three non-cancerous liver cell lines. Compared to the non-cancerous liver cell lines, the three liver cancer cell lines had barely detectable levels of PFN1 protein expression (Supplementary Figure S9). Of the remaining two PFN1 positive liver cancer cell lines, one was reported to be of endothelial origin (SK-HEP-1) [16]. An aberrant reduction of PFN1 expression appears to be a common phenomenon of advanced HCC and could contribute to the poor disease-free survival.

**Table 2:** Clinical characteristic of the patients

Characteristic	No. of patients (%)
**Total Number**	*n* = 86
**Gender**	
Male	74 (86.0)
Female	12 (14.0)
**Age (years)**	
Mean ± SEM	54.6 ± 8.6
Range	37–73
**Tumor Stage**	
T1	14 (16.3)
T2	35 (40.7)
T3	34 (39.5)
T4	3 (3.5)

## DISCUSSION

We have discovered that GUTK, a natural compound isolated from the fruits of *Garcinia yunnanensis* [15], strongly inhibits hepatic cancer cell migration and invasion without affecting cell survival and proliferation *in vitro*, and effectively suppresses lung metastasis of hepatic cancer cells without any apparent toxicity *in vivo*. To our knowledge, this is the first report of an anti-metastatic property of GUTK.

Proteomics data suggested that PFN1 is the main mediator of the anti-metastatic action of GUTK. Both the mRNA and protein levels of PFN1 were markedly increased in response to GUTK. Over-expression of PFN1 mimicked the suppressive effect of GUTK on cell motility *in vitro* and metastasis *in vivo*. Consistently, the effect of GUTK on migration and invasion was markedly diminished in HepG2 cells where PFN1 was over-expressed, suggesting that exogenously expressing PFN1 is sufficient to make GUTK action redundant. Similarly, knocking-down PFN1 diminished the effect of GUTK on cell migration and invasion, indicating that the manifestation of GUTK action requires the presence of sufficient PFN1. The importance of the induction of PFN1 by GUTK is further supported by the evidence that a decrease in PFN1 expression by siRNA promotes HCC cell motility.

The actin cytoskeleton maintains eukaryotic cell structure and integrity, and provides metastatic cancer cells the required force to move through tissues [17, 18]. PFN1 is an evolutionarily conserved actin binding protein responsible for the addition of GTP-bound actin monomers to the bark end of filaments [17, 18]. Intuitively, an increase in PFN1 expression by transfection or GUTK treatment would expect to increase the expression of F-actin cytoskeleton. The somewhat surprising results of a decrease in F-actin with unperturbed G-actin levels prompted us to examine the expression of proteins responsible for nucleation, branching and polymerization of the actin filaments in response to GUTK treatment. GUTK treatment in HCC cells led to a decrease in F-actin expression levels, which was accompanied by the inactivation of Rho family GTPases, as evidenced by the reduction in the phosphorylation of Rac1/Cdc42, as well as the reduction of their target proteins WAVE-2 and N-WASP. Exogenously expressing PFN1 also resulted in the inactivation of the Rho family of GTPases with a decrease in WAVE-2 and N-WASP expression. Protein levels of the actin-binding protein complex Arp2/3, which forms an actin nucleation site for branching [19], were also reduced upon the induction of PFN1. Diap1 is a member of the mammalian diaphanous-related formin (mDia/diap) subfamily, and a downstream effector of the Rho family of GTPases [20, 21]. The expression of Diap1 was also down-regulated when PFN1 was over-expressed or treated with GUTK. Similarly, protein expression levels of cortactin, which recruit Arp2/3 to actin filaments [22], were also decreased in response to PFN1 over-expression or GUTK-treatment. These results suggest that, besides the addition of GTP-bound actin to the bark end of actin filament, PFN1 also plays a role in the homeostasis of actin polymerization through direct or indirect regulation of the GTPases-WASP/WAVE pathways. We propose that in HCC cells low levels of PFN1 and up-regulation of the GTPases-WASP/WAVE pathways favors formation of F-actin, cell motility and metastasis. An increase in PFN1 dampens the GTPases-WASP/WAVE pathways either directly or through disrupting the existing balance in HCC cells, or a combination of both, leading to low F-actin formation and a decrease in cell motility and metastasis. Further study will be needed to determine the effect of PFN1 and GUTK on capping proteins that terminate the filament ends, and proteins for debranching and depolymerization, as they can in theory influence the dynamic homeostasis between F-actin and G-actin. Since profilin1-phosphoinositide interaction has been shown to regulate cell migration independent of its actin-related activity [23], its significance in HCC also needs to be verified. In contrast, although GUTK-treatment led to a reduction in the protein expression levels of cofilin, which cause actin filament-end depolymerization [24], yet cofilin levels were unaffected upon PFN1 over-expression, therefore, it is likely that GUTK negatively controls cofilin expression independently upon the induction of PFN1.

A reduction in PFN1 protein levels has been reported in cancers of the breast [25], esophagus [26], pancreas [27], larynges [28], and bladder [29]. In renal carcinoma, PFN1 levels are increased in the tumor stromal cells, but in normal tissue it is the renal tubule where PFN1 was stained [30]. An early study has shown that there is a decrease in PFN1 protein expression in liver cancer specimens, without referring to any of the clinical stages [31]. The present study demonstrates for the first time a negative association between PFN1 levels and the stage of HCC in 86 HCC patients. Importantly, aberrantly reduced PFN1 levels in HCC patients predict poor survival. In isolation, this prediction reflects merely an association between low protein levels of PFN1 and the survival rates of the patients, which could be due to the advanced stage of HCC rather than PFN1 protein levels *per se*. However, taken in conjunction with our findings that the protein levels of PFN1 are inversely associated with the HCC cell motility and metastasis *in vitro* and *in vivo*, we propose that aberrantly reduced PFN1 in advanced HCC plays a causal role in contributing to regional and distant metastasis. We suggest that PFN1 needs to be taken into consideration in future studies of HCC metastasis.

In conclusion, we identified PFN1 as a new biomarker and potential therapeutic target of advanced HCC. The natural compound GUTK isolated from *Garcinia yunnanensis* is a novel and potent anti-HCC metastasis agent without apparent toxicity. The inhibitory effect of GUTK on HCC metastasis is mainly mediated through the restoration of the aberrantly reduced PFN1 protein expression in HCC cells. The potential impact of these findings in the clinical management of advanced HCC warrants further evaluation.

## MATERIALS AND METHODS

### Chemical compounds

GUTK (CAS 929695-89-6, C_38_H_50_O_6_, MW: 602.80) was isolated from *Garcinia yunnanensis* as previously described [15]. Its structure was determined using ^1^H-NMR and ^13^C-NMR spectral analysis (Figure 1A), and its purity was more than 98% as determined by high pressure liquid chromatography analysis. A 20 mM stock of GUTK was dissolved in DMSO, and further dilution in culture medium was prepared prior to testing.

### Cell cultures and transfection

Normal human hepatic cell line THLE-3 (Cat. #: CRL-11233) was obtained from American Type Culture Collection. Human hepatic cancer cell lines HepG2 (Cat. #: TCHu72), Hep3B (Cat. #: TCHu106), HuH-7 (Cat. #: TCHu182), Li-7 (Cat. #: TCHu183), PLC/PRF/5 (Cat. #: TCHu119), SK-HEP-1 (Cat. #: TCHu109) and normal human hepatic cell line L-02 (Cat. #: GNHu6) were obtained from the Chinese Academy of Science Committee Type Culture Collection Cell Bank. HepG2 cell line was transfected with PFN1 vector or control vector, and selected in G418 for stable HepG2-PFN1 and HepG2-Control, respectively (Shanghai Gemechem Co Ltd, Shanghai, China). Normal human hepatic cell line MIHA was provided by the Chinese University of Hong Kong. HepG2, HepG2-PFN1 (PFN1-over-expressed HepG2), Hep3B, SK-HEP-1 and PLC/PRF/5 cells were cultured in MEM (Cat. #: 10-010-CVR; Corning, Manassas, VA, USA) supplemented with 10% FBS (Cat. #: 10099-141; Invitrogen, Carlsbad, CA, USA). Li-7 and L-02 cells were cultured in RPMI-1640 (Cat. #: 10-040-CVR; Corning) supplemented with 10% FBS. HuH-7 and MIHA cells were cultured in DMEM (Cat. #: 10-013-CVR; Corning) supplemented with 10% FBS. THLE-3 cells were cultured in BEGM (Cat. #: cc-3171; Lonza, walkersuille, MD, USA) supplemented with 5 ng/ml epidermal growth factor (Cat. #: AF-100-15; PerpoTech, Rocky Hill, NJ, USA), 70 ng/ml phosphoethanolamine (Cat. #: P05030; Sigma, St. Louis, MO, USA) and 10% FBS. All cell lines were maintained in a humidified atmosphere of 95% air and 5% CO_2_ at 37°C.

Stealth RNAi^™^ siRNAs for PFN1-1 (5′-GCUA GUCCUGCUGAUGGGCAAAGAA-3′ and 5′-UUCUUU GCCCAUCAGCAGGACUAGC-3′); and PFN1-2 (5′-CAC GGUGGUUUGAUCAACAAGA AAU-3′ and 5′-AUUUC UUGUUGAUCAAACCACCGUG-3′) and for control were transfected using Lipofectamine^TM^ RNAiMAX (Cat. #: 13778-075; Invitrogen) according to the manufacturer's protocol. Plasmid transfection was performed using X-tremeGENE HP DNA Transfection (Cat. #: 06 366 236 001; Roche, Penzberg, Germany) according to the manufacturer's protocol.

### Migration and invasion assay

Cells (2 × 10^4^ cells/well) were added to the upper chamber of the transwell (8 μM pore size, Cat. #: PIEP12R48; Millpore, Schwalbach, Germany) after treated with GUTK for 15 min in 100 μl medium with 0.1% BSA; and 600 μl of complete medium was added to the lower chamber. The chamber was then cultivated in 5% CO_2_ at 37°C for 24 h or 48 h. Non-migrated cells in the upper chamber were removed, and migrated cells were fixed in 4% paraformaldehyde (PFA) and stained with 0.1% crystal violet. The migration rate and inhibition rates (%) were quantified by counting the migration cells in 5 random fields under an inverted microscope (Olympus, Japan). The invasion assay was the same with migration assay except that the upper chamber surface of the transwell membrane was coated with 20 μg Matrigel (Cat. #: 356237, Phenol Red-free; BD Biosciences, Bedford, MA, USA) for 30 min before the cells were seeded and the cell suspension for the upper chambers were 2.5 × 10^5^ cells.

### Western blotting assay

The experimental cells were lysed in RIPA buffer (Cat. #: 9806; Cell Signalling Technology (CST), Danvers, MA, USA) (supplemented with 1 mM PMSF (Cat. #: 8553; CST). The homogenate was centrifuged at 1000 g for 5 min at 4°C and the supernatant was kept at −20°C until use. Proteins were separated by SDS-PAGE and blots were probed with combinations of primary and horseradish peroxidase conjugated secondary antibodies. For repeated immunoblotting, membranes were stripped in buffer (62.5 mM Tris with pH 6.7, 10% SDS and 0.1 M β-mercaptoethanol) for 30 min at 50°C. GAPDH served as a loading control. Primary antibodies against: phospho-Rac1/Cdc42 (p-Rac1/Cdc42) at Ser^71^ (1:500 dilution; Cat. #: 2461), Rac1/Cdc42 (1:1000 dilution; Cat. #: 4651), Diap1 (1:1000 dilution; Cat. #: 5486s), Arp2 (1:1000 dilution; Cat. #: 3128), Arp3 (1:1000 dilution; Cat. #: 4738), WAVE-2 (1:1000 dilution; Cat. #: 3659), N-WASP (1:1000 dilution; Cat. #: 4848), cofilin (1:1000 dilution; Cat. #: 3318), cortactin (1:1000 dilution; Cat. #: 3502), peroxidase-conjugated goat anti-rabbit IgG (H+L) (1:2000 dilution; Cat. #: 7074), and GAPDH (1:1000 dilution; Cat. #: 2118s) were purchased from CST; Profilin 1 (PFN1, 1:1000 dilution; Cat. #: ab133529) was purchased from Abcam (Cambridge, UK).

### Immunofluorescence assay

HepG2 cells (1.5 × 10^5^ cells/well) were allowed to adhere overnight to glass coverslips in a 6-well plate. After treatment, cells were fixed with 4% PFA for 30 min at room temperature. Fixed cells were incubated with 0.3% Triton X-100 in PBS, and blocked with 10% BSA in PBS for 1 h. The cells were incubated with the primary antibody against PFN1 (dilution 1:50; Cat. #: 3237; CST) or ACTN05 (C4) (1:200 dilution; Cat. #: ab3280; Abcam) overnight at 4°C followed by Alexa-Fluor 488-conjugated goat anti-rabbit IgG antibody (1:200 dilution; Cat. #: O-6381; Invitrogen) or Alexa Fluor 594 donkey anti-mouse IgG secondary antibody (1:200 dilution; Cat. #: A-21203; Invitrogen) for 1 h at room temperature. Alexa Fluor 555^®^-phalloidin (1:20 dilution; Cat. #: 8953; CST) and 4′6-diamidino-2-phenylindole (DAPI, Cat. #: P36931; Invitrogen) staining were then used to stain F-actin and nucleus, respectively. Immunofluorescence images were visualized using an inverted fluorescent microscope (Olympus, Japan).

### Animal study

Specific pathogen free BALB/c nude male mice (6 weeks old) and BALB/c male mice (6 weeks old) were purchased from the Experimental Animal Center of Chinese Academy of Science (Shanghai, China). All animal experiments were performed in accordance with a protocol approved by the Shanghai University of Traditional Chinese Medicine Committee on the Use of Live Animals for Teaching and Research and were carried out in accordance with the Guide for the Care and Use of Laboratory Animals, published by the National Institutes of Health (publication No. SCXX (HU) 2007-0005). All institutional and national guidelines for the care and use of laboratory animals were followed. For experimental lung metastasis, nude mice were randomly assigned to control and treatment groups on day 1. The body weight of each mouse was recorded every 3 days. At day 28, mice were sacrificed and lungs were harvested and fixed in Bouin's solution for photograph or 4% PFA for haematoxylin and eosin (H&E) staining. The numbers of metastasis nodules in the lung was determined by macroscopic photographs and the weight of the lung. For the orthotropic implantation, 4 × 10^6^ HepG2 cells were suspended in 100 μl DMEM and Matrigel (1:1) and then inoculated into the liver parenchyma of nude mice under 7% chloral hydrate anesthesia as previous described [32]. The health status of the mice were then monitored every two days and sacrificed 2 months later. The organs from mice, including brain, heart, lung, liver, spleen and kidney, were fixed in 4% PFA and stained by H&E. For the toxicity study, BALB/c male mice (6 weeks old) were injected with GUTK at different doses or vehicle control every second days. The body weight of each mouse was recorded every 3 days. After 28 days, mice were killed and selected tissues were fixed in 4% PFA. Serial histologic sections of the removed hearts, lungs, livers, spleens and kidneys were stained by H&E.

### Trypan blue staining

Cell viability was determined by the trypan blue exclusion assay. The experimental cells were incubated with 0.4% trypan blue in PBS for 10 min, and the dead cells were stained blue. The number of stained and unstained cells was counted using a hemocytometer and the cell viability was scored as the percentage of dead cells over both surviving and dead cells.

### Two-dimensional electrophoresis (2-DE), protein visualization and image analysis

GUTK or DMSO treated HepG2 cells were harvested and the protein concentration was determined using BioRad Dc protein Assay. A 2-DE was performed as described previously [33]. Briefly, the samples containing 150 μg of protein were diluted in a rehydration buffer, loaded onto IPG strips and rehydrated with an IPGphor II apparatus (Amersham). The isoelectric focusing was carried out in a stepwise voltage increasing manner. The gels were visualized by silver staining and the raw images were captured by using a GS-800 scanner and QuantityOne program, and subsequently analyzed by the PDQuest program (version 8.0, BioRad). The differentially expressed proteins spots were manually excised. After enzymatic digestion, the peptide samples were analyzed using a 4700 Proteomics Analyzer (TOF/TOF^™^) (Applied Biosystems). A peptide mass mapping was performed using a MASCOT program (Matrix Science, London) against Swiss-Prot database with a GPS explorer software (Applied Biosystems).

### Immunohistochemical analysis and quantification

Tissue microarrays (Cat. #: HLiv-HCC180Sur-01, Shanghai Biochip, Shanghai, China) was constructed as described previously [34]. Written informed consent was obtained from each patient, and the study protocol was approved by the Ethics and Scientific Committees of Taizhou Hospital. The tissue microarray contains 180 cylinders from 90 paired HCC and adjacent normal tissues. Sections (4 μm) were placed on slides coated with 3-aminopropyltriethoxysilane. For immunohistochemical analysis, TMA slide was first deparaffinized and then hydrated. After microwave antigen retrieval, endogenous peroxidase activity was blocked with incubation of the slides in methanol containing 3% H_2_O_2_, and nonspecific binding sites were blocked with 1% BSA. After serial incubation with PFN1 (1:1000 dilution Cat. #: ab133529, Abcam) overnight at 4°C followed by SignalStain^®^ Boost IHC Detection Reagent (HRP, Rabbit) (Cat. #: 8114, CST), the sections were developed in diaminobenzidine solution under a microscope and counterstained with hematoxylin. For quantification, immunostaining was scored according to the intensity in comparison to method and isotype controls. The average intensity of immunoreactivity was graded on a scale of 0 (none), 1+ (weak); 2+ (intermediate); and 3+ (strong). Grading was undertaken by a pathologist (C.S. L).

### Statistical analysis

Normal distributed data with equal variance were analyzed using ANOVA followed by Fisher's LSD Multiple-Comparison Test. Differences in PFN1 expression levels in HCC and adjacent tissue were tested using McNemar's Chi-Square and Wilcoxon's Ranked Sign Test at different tumor stages. Kaplan-Meier survival curves were used to describe patient survival by PFN1 expression levels in HCC and adjacent tissue, and the differences in survival were tested using the log-rank test. *P* values less than 0.05 were considered to indicate statistical significance.

## SUPPLEMENTARY MATERIALS FIGURES


