# Supplementary material for: Guttiferone K suppresses cell motility and metastasis of hepatocellular carcinoma by restoring aberrantly reduced profilin 1
Source: Oncotarget. 2016 Aug 1;7(35):56650–63. doi: 10.18632/oncotarget.10992 (PMC5302942; doi:10.18632/oncotarget.10992)
Supplement: Supplementary file 1 [file oncotarget-07-56650-s001.pdf]

Guttiferone K suppresses cell motility and metastasis of hepatocellular carcinoma by restoring aberrantly reduced profilin 1

Supplementary Materials

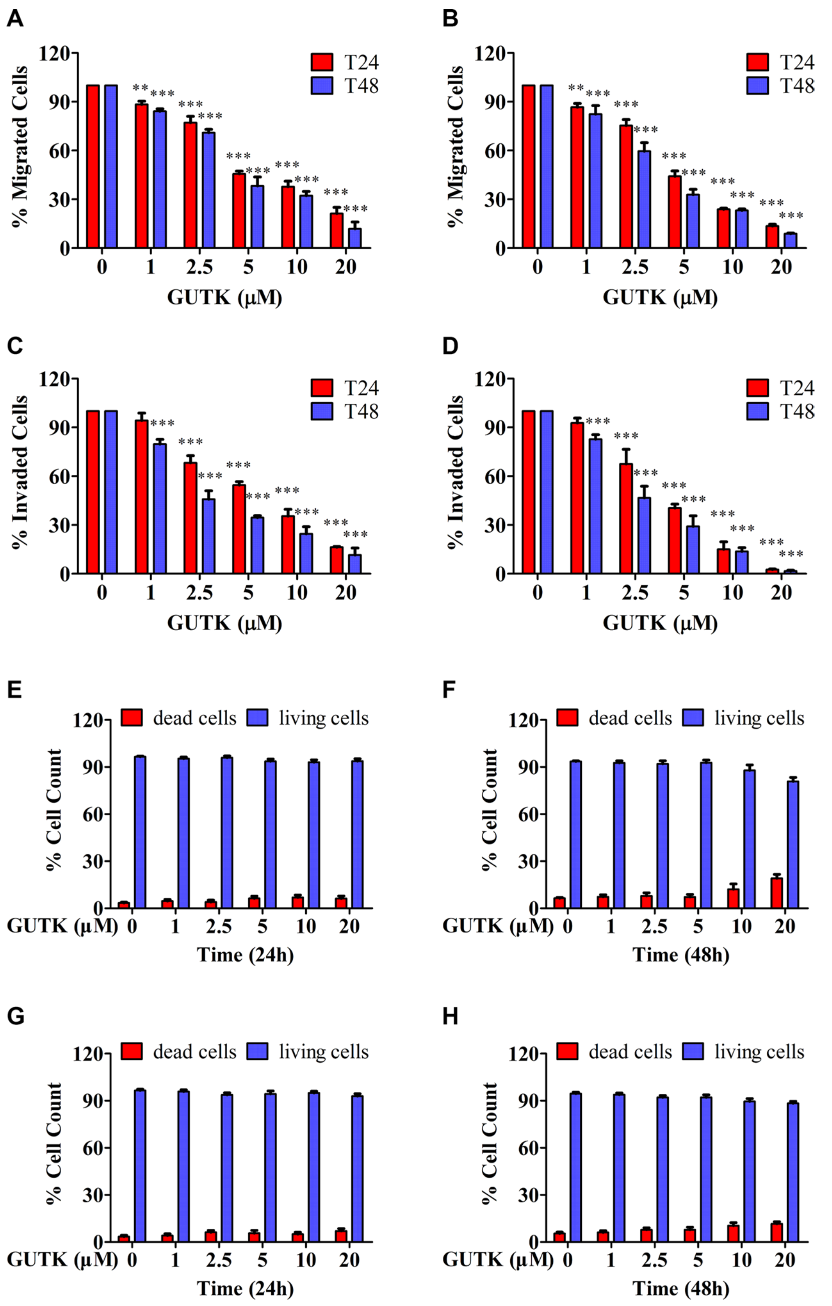

Supplementary Figure S1: GUTK suppresses HCC cell motility and metastasis *in vitro*. (**A**, **B**) Cell migration was determined after incubation with GUTK (0–20 μM) in Li-7 (A) and PLC/PRF/5 (B) cells for 24 h and 48 h. (**C**, **D**) Cell invasion was determined after incubation with GUTK (0–20 μM) in Li-7 (C) and PLC/PRF/5 (D) cells for 24 h and 48 h. (**E**, **F**) Cell proliferation was measured after incubation with GUTK (0–20 μM) in Li-7 cells for 24 h and 48 h by trypan blue exclusion. (**G**, **H**) Cell proliferation was measured after incubation with GUTK (0–20 μM) in PLC/PRF/5 cells for 24 h and 48 h by trypan blue exclusion. Data are shown as mean ± SEM; $^{**}P < 0.01$, $^{***}P < 0.001$ vs. control, $n = 3$.

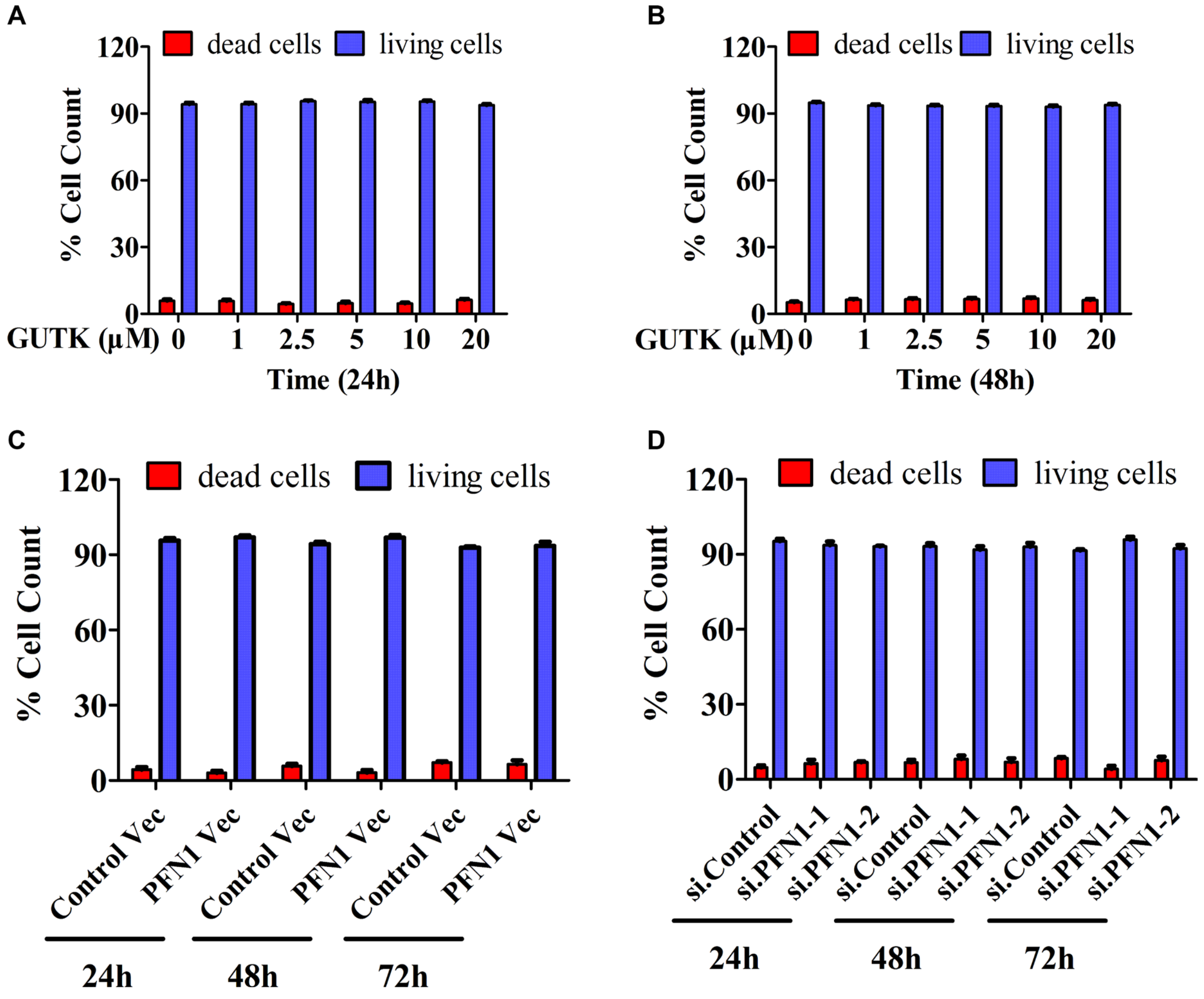

Supplementary Figure S2: The effect of GUTK and PFN1 on HCC cell viability. (**A**, **B**) Cell proliferation was measured after incubation with GUTK (0–20 μM) in HepG2 cells for 24 h and 48 h by trypan blue exclusion. (**C**, **D**) Cell proliferation was measured in HepG2 cells with transiently transfected with PFN1 vector or PFN1 siRNA for 24–72 h by trypan blue exclusion. Dead cells and living cells are expressed as the percentage of cells relative to the total amount of cells. Data are shown as means ± SEM. *n* = 3.

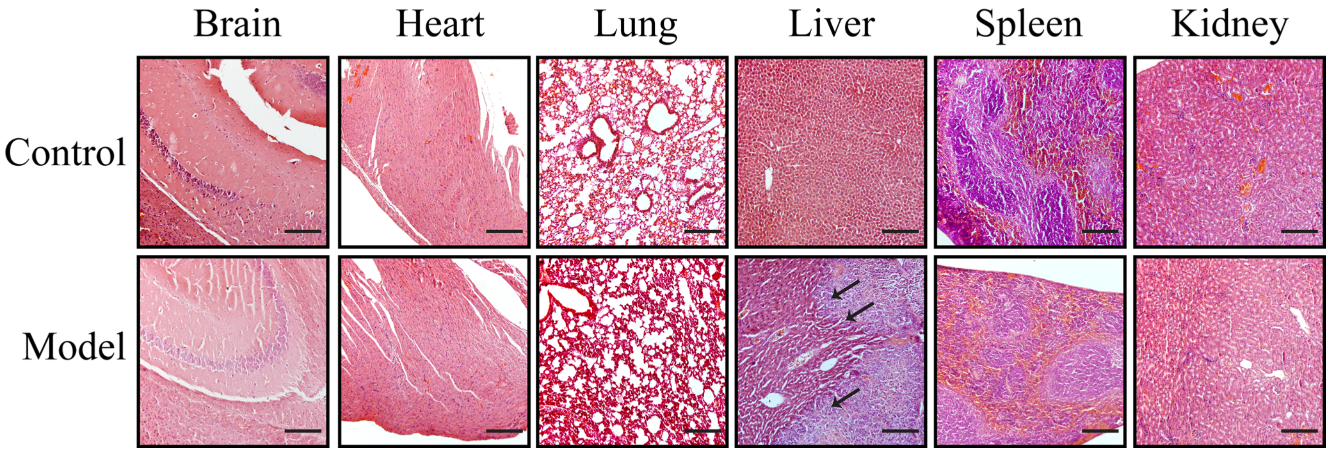

Supplementary Figure S3: Tissue analysis from mice given HepG2 cells using liver orthotropic implantation. For the orthotropic implantation, 4 × 10^6 HepG2 cells were suspended in 100 μl DMEM and Matrigel (1:1) and then inoculated into the liver parenchyma of nude mice under 7% chloral hydrate anesthesia. The health status of the mice were then monitored every two days and sacrificed 2 months later. The organs from mice, including brain, heart, lung, liver, spleen and kidney, were fixed in 4% PFA, and sectioned for H&E staining. Arrow points to the tumor cells. *n* = 6.

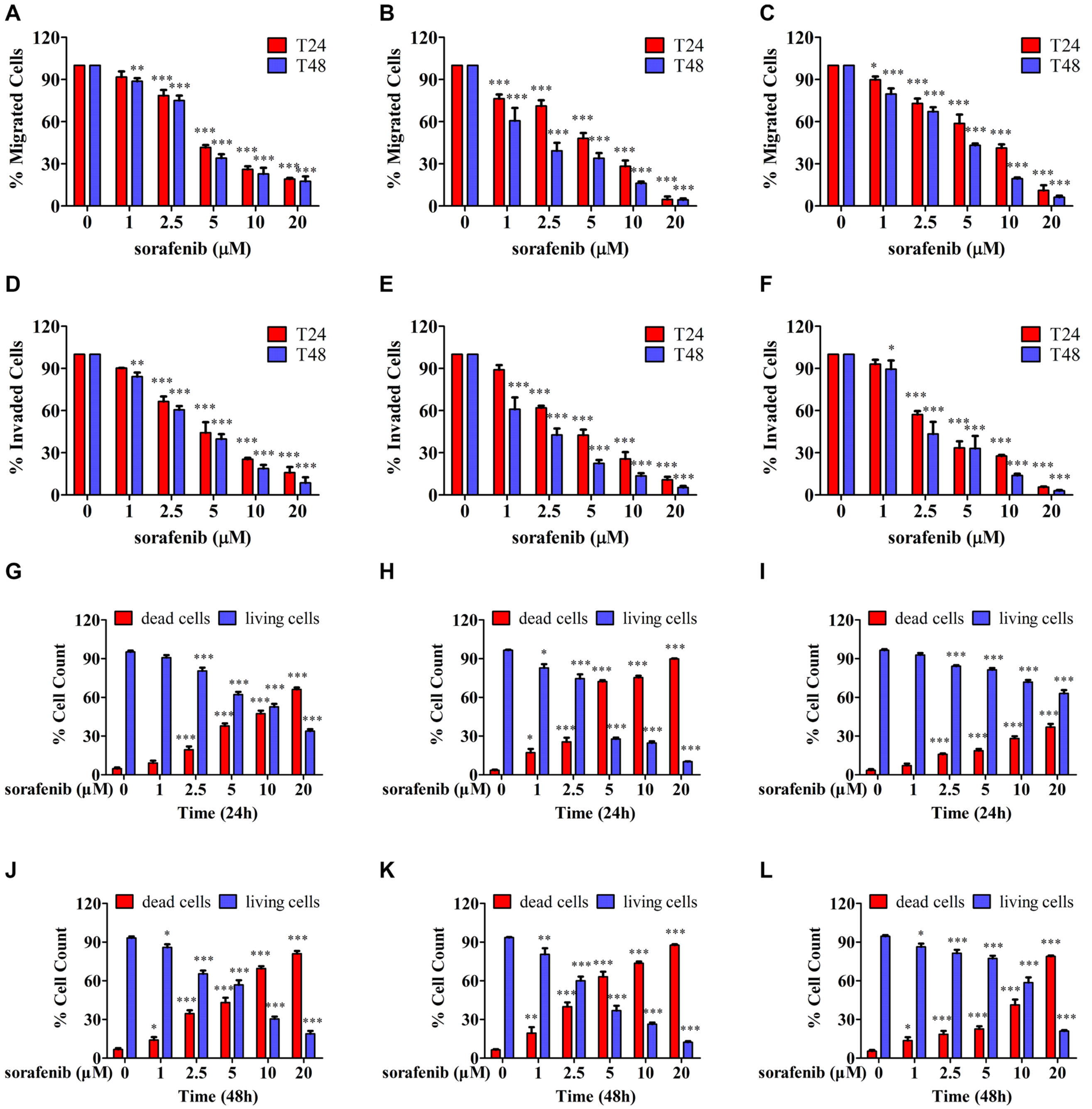

Supplementary Figure S4: Sorafenib suppresses HCC cell motility and metastasis *in vitro*. (**A**–**C**) Cell migration was determined after incubation with sorafenib (0–20 μM) in HepG2 (A), Li-7 (B) and PLC/PRF/5 (C) cells for 24 h and 48 h. (**D**–**F**) Cell invasion was determined after incubation with GUTK (0–20 μM) in HepG2 (D), Li-7 (E) and PLC/PRF/5 (F) cells for 24 h and 48 h. (**G**–**I**) Cell proliferation was measured after incubation with sorafenib (0–20 μM) in HepG2 (G), Li-7 (H) and PLC/PRF/5 (I) cells for 24 h by trypan blue exclusion. (**J**–**L**) Cell proliferation was measured after incubation with GUTK (0–20 μM) in HepG2 (J), Li-7 (K) and PLC/PRF/5 (L) cells for 48 h by trypan blue exclusion. Data are shown as mean ± SEM; *$P < 0.05$, **$P < 0.01$, ***$P < 0.001$ vs. control, $n = 3$.

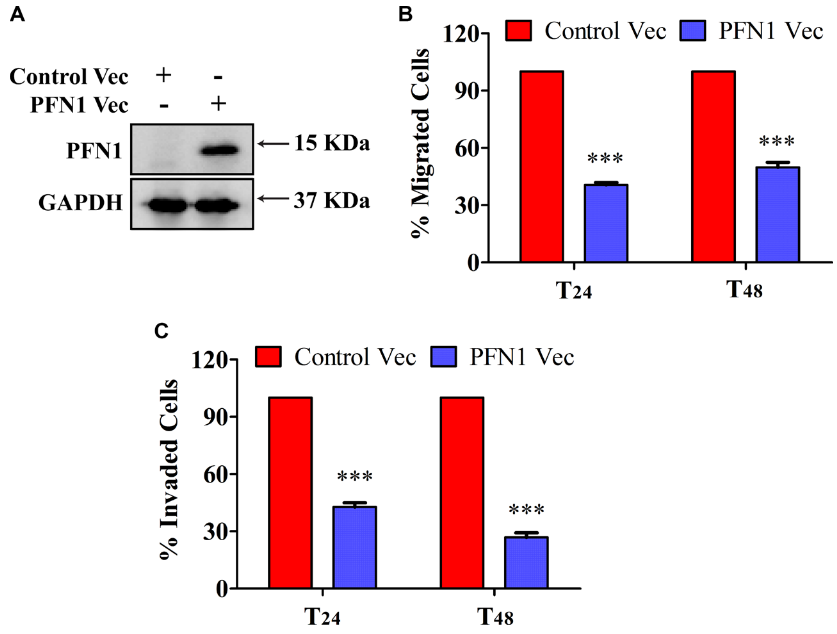

Supplementary Figure S5: The effect of PFN1 overexpression on HepG2 cell motility. (**A**) Western blotting analysis for protein expression of PFN1 and GAPDH were determined in HepG2 cells with stable-transfected PFN1 or control vector. (**B**, **C**) Cell migration activity and cell invasion activity were determined in HepG2 cells with or without stable transfected with PFN1 vector for 24 h and 48 h. Data are shown as means ± SEM; ****P* < 0.001 compared with Control Vector. *n* = 3.

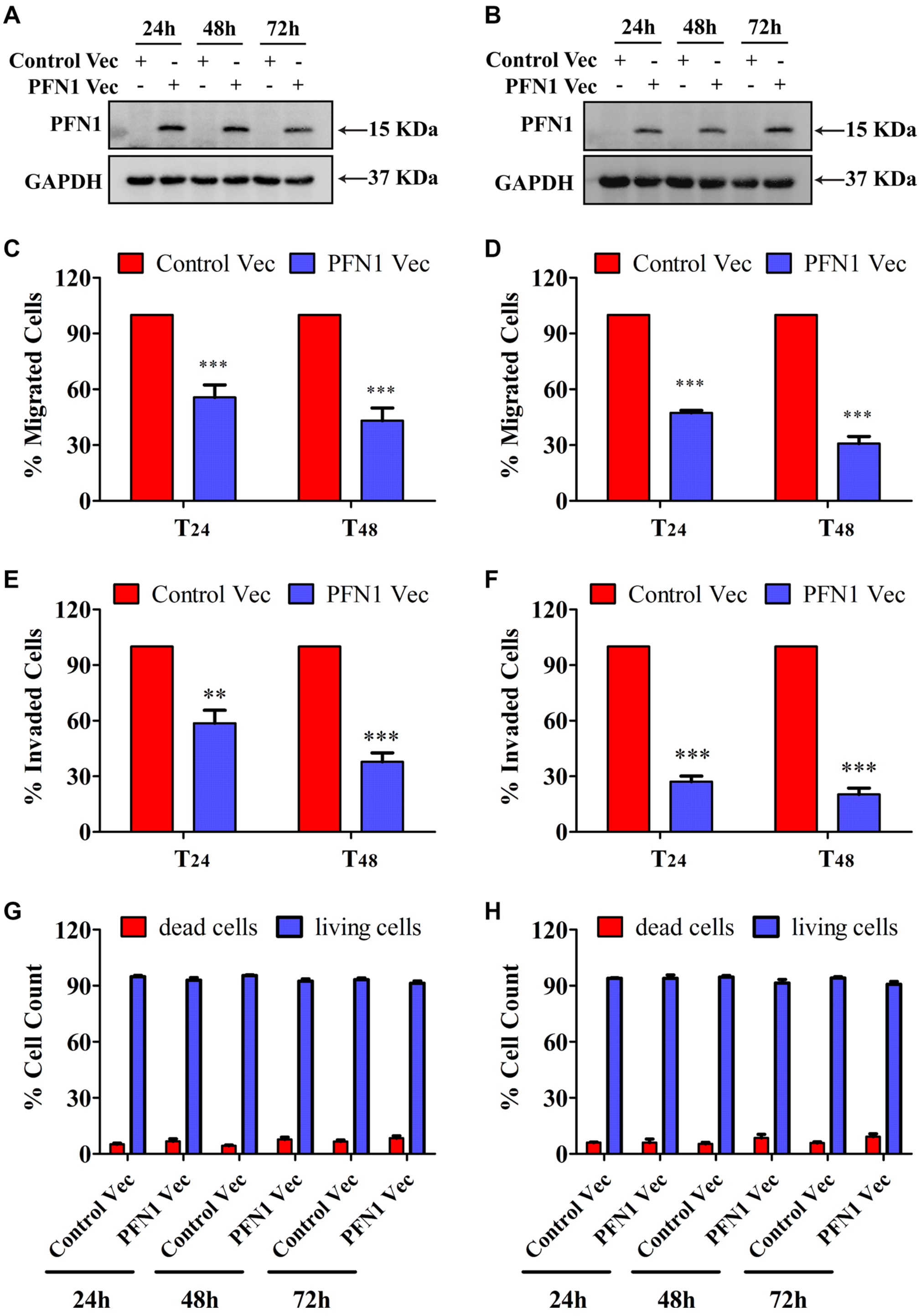

Supplementary Figure S6: The effect of PFN1 overexpression on Li-7 and PLC/PRF/5 cell motility. (**A**, **B**) PFN1 in Li-7 (A) and PLC/PRF/5 (B) cells transiently transfected with PFN1 Vec or control Vec for 24–72 h, cells were then lysed and indicated proteins were analyzed by SDS-PAGE/western blotting. GAPDH served as a loading control. (**C**, **D**) Cell migration was determined in Li-7 (C) and PLC/PRF/5 (D) cells transiently transfected with PFN1 or control vector for 24 h and 48 h. (**E**, **F**) Cell invasion was determined in Li-7 (E) and PLC/PRF/5 (F) cells transiently transfected with PFN1 or control vector for 24 and 48 h. (**G**, **H**) Cell proliferation was measured in Li-7 (G) and PLC/PRF/5 (H) cells with transiently transfected with PFN1 vector for 24–72 h by trypan blue exclusion. Dead cells and living cells are expressed as the percentage of cells relative to the total amount of cells. Data are shown as mean ± SEM; $^{**}P < 0.01$, $^{***}P < 0.001$ vs. cells transfected with control vector. $n = 3$.

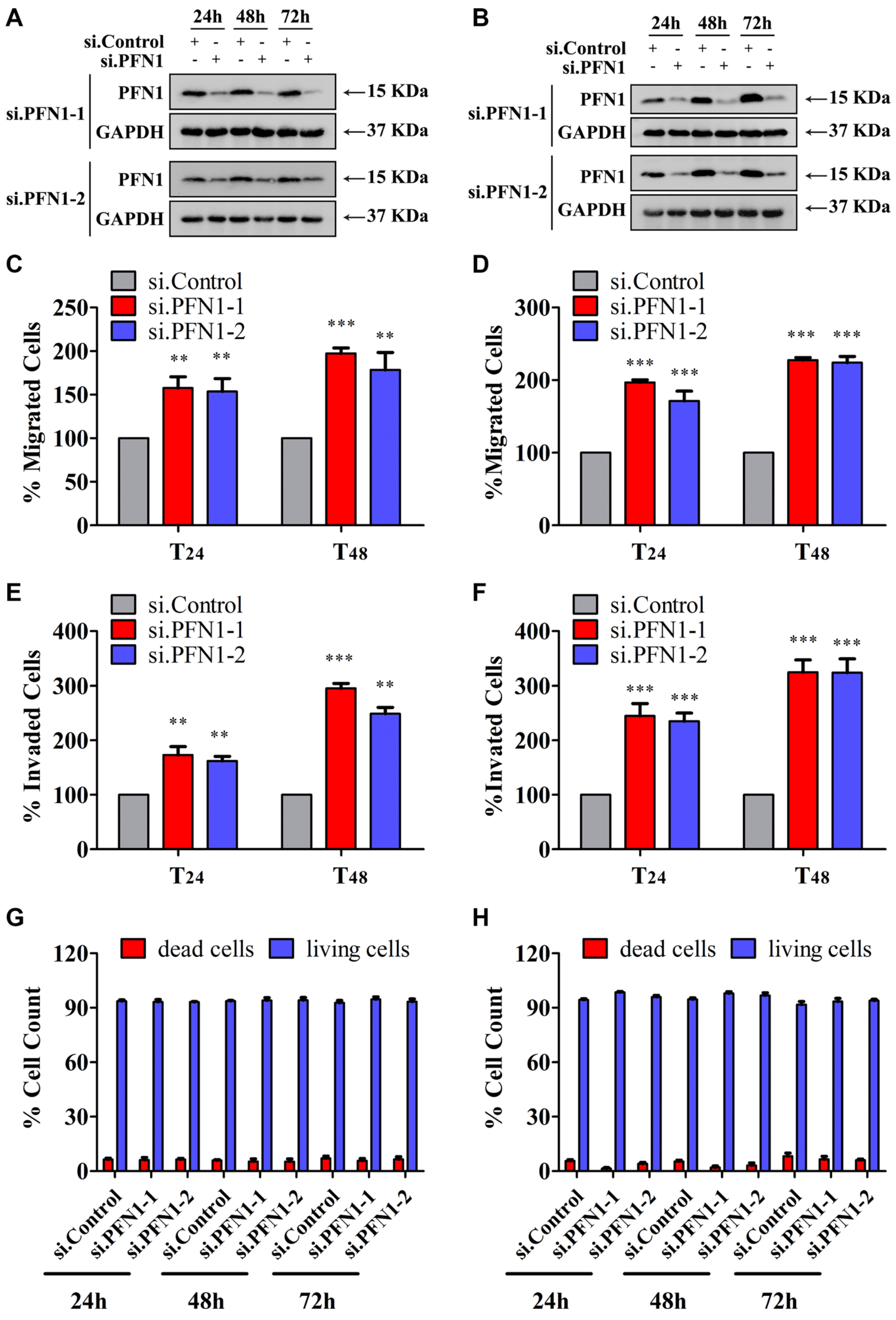

Supplementary Figure S7: Silenced expression of PFN1 by PFN1-specific siRNA on the motility of Li-7 and PLC/PRF/5 cells. (**A**, **B**) PFN1 protein expression levels in Li-7 (A) and PLC/PRF/5 (B) cells transiently transfected with two individual PFN1 siRNAs or control siRNA for 24–72 h were determined by SDS-PAGE/western blot analysis. (**C**, **D**) Cell migration was determined in Li-7 (C) and PLC/PRF/5 (D) cells transiently transfected with PFN1 siRNAs or control siRNA for 24 h and 48 h. (**E**, **F**) Cell invasion was determined in Li-7 (E) and PLC/PRF/5 (F) cells transiently transfected with PFN1 siRNAs or control siRNA for 24 h and 48 h. (**G**, **H**) Cell proliferation was measured in Li-7 (G) and PLC/PRF/5 (H) cells transiently transfected with PFN1 siRNAs for 24–72 h by trypan blue exclusion. Dead cells and living cells are expressed as the percentage of cells relative to the total amount of cells. Data are shown as mean ± SEM; $^{**}P < 0.01$, $^{***}P < 0.001$ vs. cells transfected with control vector. $n = 3$.

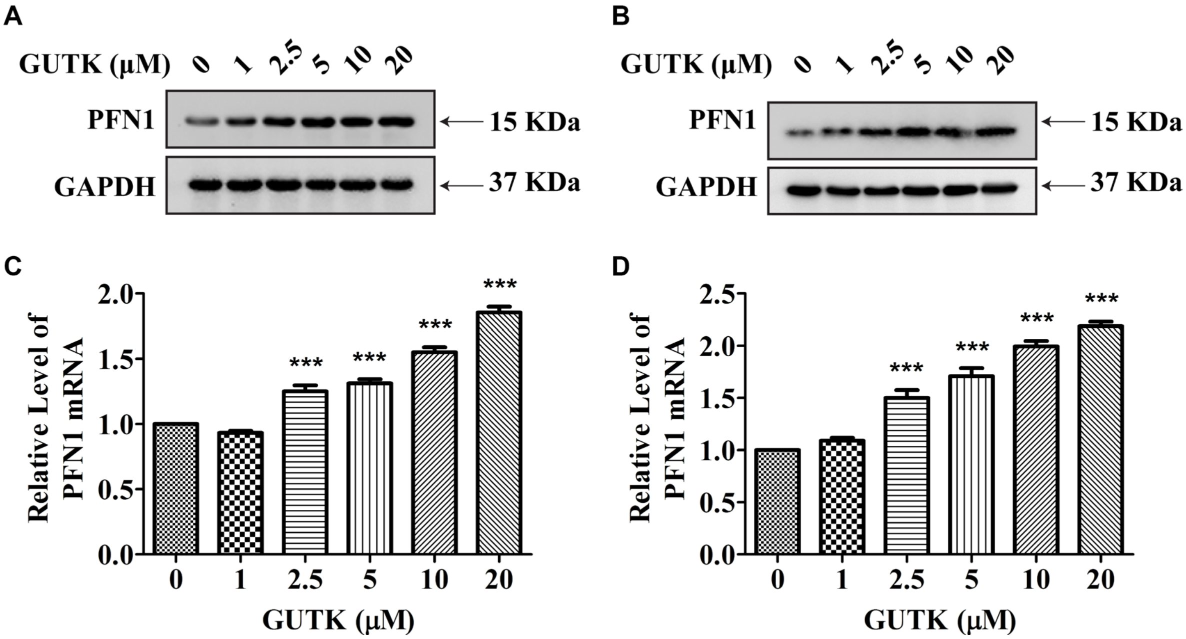

Supplementary Figure S8: GUTK induces increases in the mRNA and protein expression levels of PFN1 in Li-7 and PLC/PRF/5 cells. (**A**, **B**) Protein expression levels of PFN1 and GAPDH were measured by SDS-PAGE/Western blot analysis after treatment with GUTK (0–20 μM) for 24 h in Li-7 (A) and PLC/PRF/5 (B) cells. (**C**, **D**) mRNA expression levels of PFN1 and GAPDH were measured by qPCR after treatment with GUTK (0–20 μM) for 24 h in Li-7 (C) and PLC/PRF/5 (D) cells. Data are shown as mean ± SEM; ****P* < 0.001 vs. control. *n* = 3.

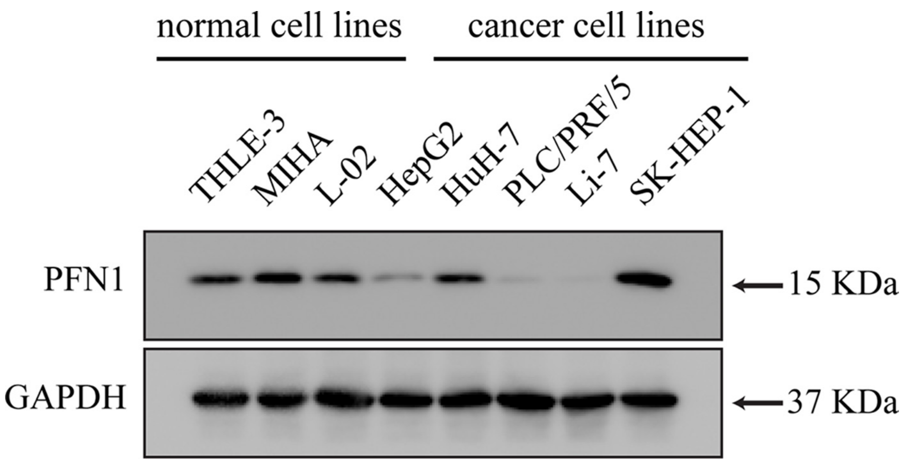

Supplementary Figure S9: The PFN1 protein levels in non-cancerous and cancer-derived liver cell lines. Western blotting analysis for protein expression of PFN1 and GAPDH in THLE-3, MIHA, L-02, HepG2, HuH-7, PLC/PRF/5, Li-7, and SK-HEP-1 cells. *n* = 3.
